# Anaesthetic Management of a Child With Severe Spastic Quadriplegic Cerebral Palsy: A Case Report

**DOI:** 10.7759/cureus.109780

**Published:** 2026-05-27

**Authors:** Manish Rai, Rajeshwari Gore, Piyush Setu

**Affiliations:** 1 Anaesthesiology, Max Healthcare, Delhi, IND; 2 Pharmacology and Therapeutics, Sharda School of Medical Sciences and Research, Greater Noida, IND

**Keywords:** anaesthetic management, atracurium, cerebral palsy, difficult venous access, spastic quadriplegic cerebral palsy

## Abstract

Patients with cerebral palsy are known to present with numerous anaesthetic challenges due to spasticity, joint deformities, cognitive dysfunction, and behavioural abnormalities. Our patient was an eight-year-old girl with severe spastic quadriplegic cerebral palsy (SQCP) posted for cataract surgery with IOL (intraocular lens) placement under general anaesthesia. A thorough preanaesthetic checkup (PAC) was performed, after which the family members were counselled, and appropriate medications based on the patient’s condition were prescribed to prepare her for the anaesthetic and surgical procedures. We encountered initial challenges with cannulation and patient positioning, but apart from that, we successfully managed the patient throughout the surgical procedure, and recovery was smooth. We conclude that every patient may respond differently, but meticulous assessments and comprehensive planning, along with a multidisciplinary approach, can certainly ensure a smooth and comfortable experience for the patient and pose fewer challenges for the anaesthesiologist.

## Introduction

Cerebral palsy (CP) is characterized by a spectrum of permanent but non-progressive central nervous system disorders. It is one of the most common causes of neurodisability in children [1]. The global prevalence of CP is reported to be 2.1 per 1,000 live births. Suggested causes of CP include a lack of proper oxygen supply to the foetus, infections, coagulopathies, and genetics [2]. The affected areas of the brain include the motor cortex, basal ganglia, and cerebellum, which lead to motor dysfunction that impacts posture, movement, and muscle tone. Impairment in these areas further leads to spasticity and affects the communication skills, behaviour and intellect of the child. Approximately 80% of the children with CP will have spasticity as the main dysfunction [3]. One of the types of CP is spastic quadriplegic cerebral palsy (SQCP), characterized by severe stiffness and weakness of all four limbs. These patients pose challenges during anaesthetic management because of the stiffness in the limbs, which makes proper positioning and intubation difficult; establishing intravenous cannulation could be arduous due to the non-prominent veins, and there is always a risk of aspiration due to the excessive oral secretions [4-6]. We report the anaesthetic management of a child with SQCP and the challenges we faced during positioning and administering medications in the perioperative period.

## Case presentation

An eight-year-old female patient with SQCP presented to the Ophthalmology department with poor vision and no response to light or sound. She was diagnosed with bilateral advanced cataracts and posted for cataract surgery with IOL (intraocular lens) placement under general anaesthesia.

Pre-anaesthesia checkup (PAC)

During the PAC, we noted that there was significant growth retardation, and the patient weighed 9 kg, which was less than the 3rd percentile for her age. Further evaluation showed that the patient was disoriented, unable to communicate with signs, and did not recognize the parents. On oral examination, we found that she had excessive oral secretions.

**Figure 1:**
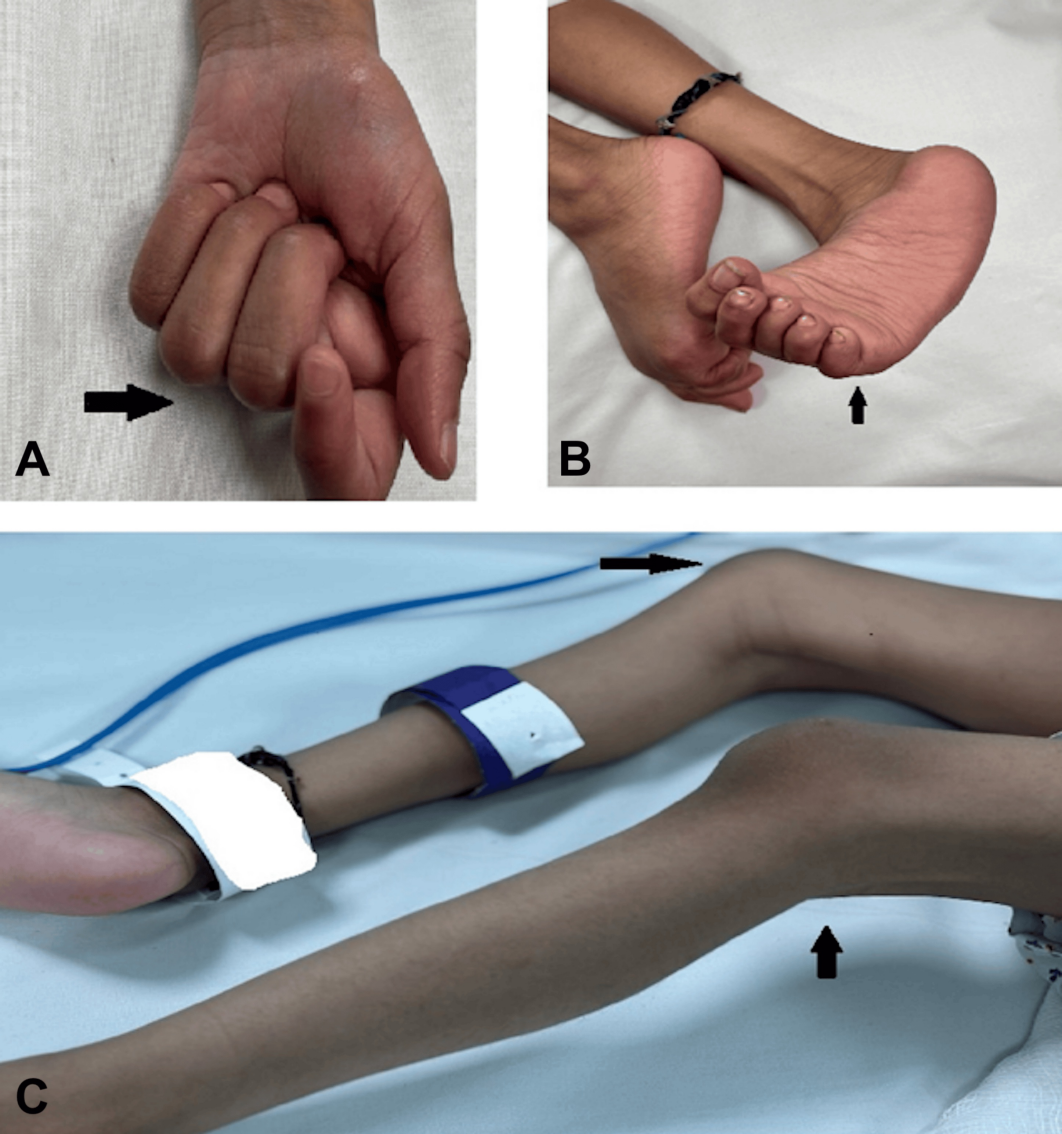
Spasticity in the limbs (A-C)

Physical examination revealed severe stiffness in both upper and lower limbs, with a limited range of motion in all extremities due to increased muscle tone and spasticity, as shown in Figure 1. There was a significant gross motor developmental delay. Her cardiac system appeared normal. An echocardiogram showed the ejection fraction of 55%. No abnormalities were detected during the abdominal examination. The patient was advised to get an MRI and a neurological opinion.

**Figure 2:**
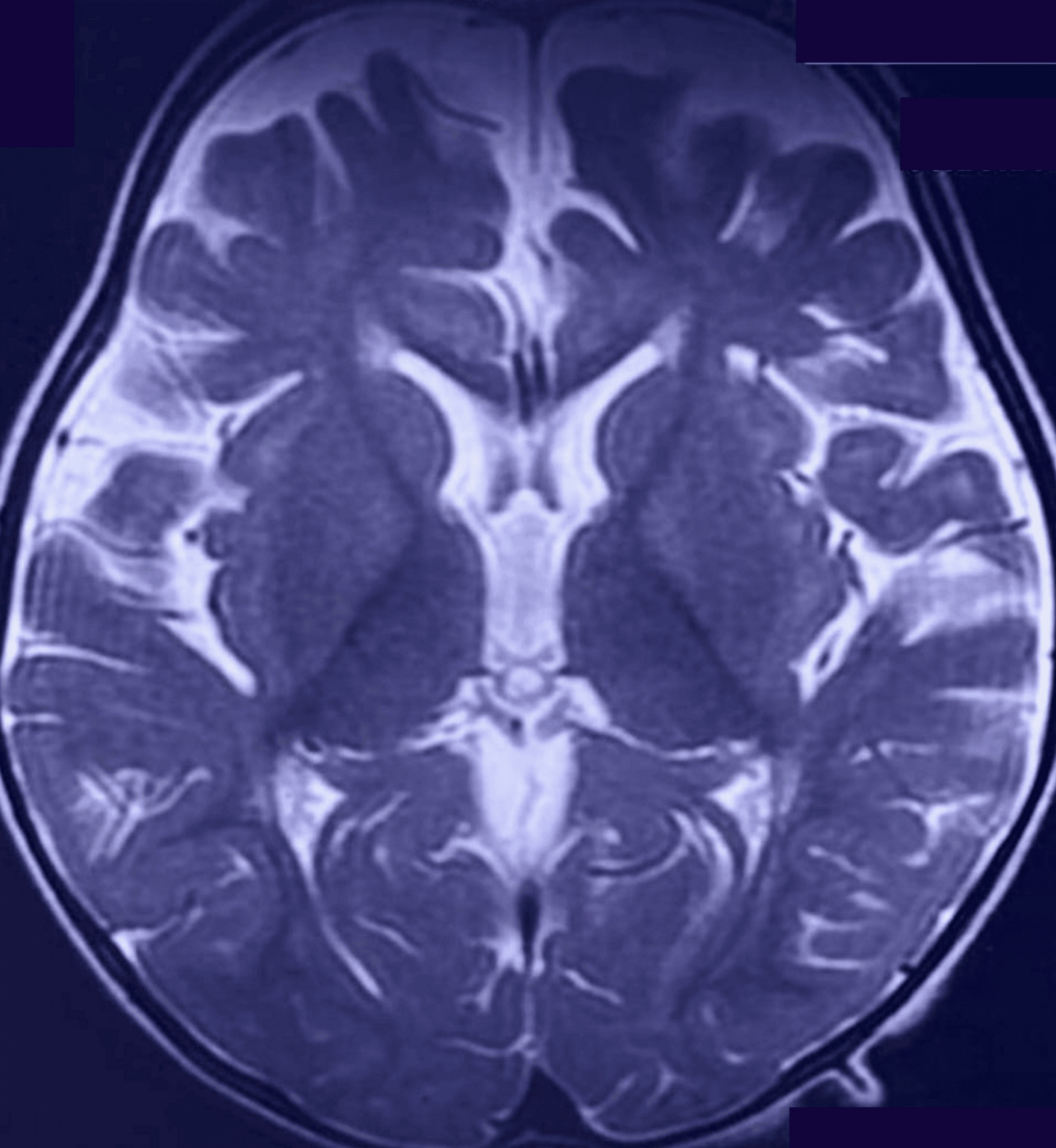
MRI axial view

On MRI, generalized prominence of cortical sulci, sulcal space, sylvian fissure, and basal cisterns with disproportionate mild ventricular prominence suggestive of generalized cerebral atrophy was noted, as shown in Figure 2. Mild leucomalacic changes were also seen near the frontal horn of bilateral lateral ventricles. On respiratory examination, it was found that the child had scattered B/L rhonchi with conducted sounds. A chest X-ray showed bilateral upper lobe pneumonitis. Nebulization with a combination of levosalbutamol + ipratropium bromide, one respule of 2.5 ml 6-8 hourly, antibiotic syrup of amoxicillin + clavulanic acid 25 mg/kg/d in two divided doses and supportive therapy like an upright position during meals to prevent silent aspirations and steam inhalations thrice a day were advised for five days to prepare the patient for the operative procedure. Routine blood work was within normal limits (CBC, coagulation profile, KFT (kidney function test), LFT (liver function test), thyroid profile, and blood sugar).

Birth history revealed that the patient was a full-term LSCS (lower segment caesarean section) delivery with low birth weight (2.1 kg), meconium-stained liquor, birth asphyxia, postnatal cyanotic spells, and NICU admission for four days. Neonatal sepsis and TORCH infections were ruled out. The child developed seizures until four years of age. Currently, the patient is not taking any medication and has no known allergies.

We faced a challenge in properly positioning the patient on the operating table owing to the severe spasticity in the patient's limbs; hence, it was decided that we keep the positioning as natural as possible: knees were supported with bolsters, hands were rested on the chest, and the head ring was cushioned with extra layers of sheets to support the neck, which was otherwise suspended. Cannulation was demanding, as the veins were not prominent, but once the patient was induced, it was achieved by a 24-gauge cannula on the dorsum of the left hand; no USG (ultrasonography) was required. Airway intubation was also complicated due to the spasticity but was achieved by securing the airway with a classic LMA #2, which was preferred over intubation due to the short course of the surgery of one hour.

For inducing the patient, we used inhalational sevoflurane and the antisialagogue effect was achieved by IV glycopyrrolate 0.004 mg/kg. Analgesia was obtained by IV fentanyl 2 μg/kg. Maintenance was done with oxygen, nitrous oxide, and sevoflurane. Skeletal muscle relaxation was obtained by injectable atracurium 0.2 mg/kg. Haemodynamic variables remained stable throughout the surgery. The Bispectral Index (BIS) was not used, and the patient's temperature was not monitored. The patient was well covered with extra sheets. Regarding the ventilatory parameters, tidal volume was 90 ml/min, and frequency was 18 breaths/min. With this, ETCO₂ was maintained in a range of 35-40 mmHg.

Once the phacoemulsification cataract surgery with IOL placement was completed, the neuromuscular blockade was reversed by injecting neostigmine 0.05 mg/kg along with glycopyrrolate 0.008 mg/kg. Following an uneventful reversal and extubation, the patient was transferred to the recovery room. She remained haemodynamically stable for four hours, reporting no pain, nausea, or vomiting. After this, she was given oral fluids and voided spontaneously. The patient was discharged six hours postoperatively after meeting all discharge criteria.

## Discussion

We report a case of an eight-year-old female patient with SQCP, who was posted for cataract surgery with IOL placement under general anaesthesia. In SQCP, all four limbs show spasticity; therefore, positioning the patient under anaesthesia is quite challenging and should be done with extreme care. Due to neck rigidity, we anticipated a difficult intubation, which we mitigated by elevating the neck with adequate cushioning and opting for an LMA, which helped us achieve adequate ventilation in the patient. Bag-and-mask ventilation was adequate without difficulty. Another possible complication could have been aspiration due to excessive oral secretions in these patients; an adequate dose of an antisialagogue, such as glycopyrrolate, during induction and recovery shielded us from this complication. Dose titration of muscle relaxants is necessary due to reduced muscle mass, which increases their susceptibility to toxicity. Monitoring and maintenance of core temperature are also vital, as they are prone to hypothermia.

To start with, we found that intravenous cannulation was quite difficult in this patient due to the lack of any prominent veins. Reports do confirm that CP is a risk factor for difficult venous access [7]. Among the various approaches to improving visibility and access to the veins, some reports suggest topical application of a eutectic mixture of local anaesthetics (EMLA), alone or with a nitroglycerin ointment. They are indicated to improve the vein visibility due to local vasodilatation [8,9].

In our patient, we chose sevoflurane for induction, as it has proven efficacy, produces faster action, and is non-pungent, making it ideal for administration in children with an LMA. It offers an added advantage: it does not stimulate the sympathetic system or cause any airway irritation [10,11].

For muscle relaxation, we used atracurium due to its unique Hofmann elimination and rapid action, thereby preventing residual muscle relaxant effects. Atracurium provided effective and adequate muscle relaxation in our patient. These findings were contrary to a report by Panwar S, who described a case of CP in a 15-year-old male patient who was anaesthetized for debridement and external fixator application of a subtrochanteric femur fracture. She reported that atracurium was inadequate for maintaining immobility, and they had to switch the skeletal muscle relaxant to achieve an adequate response [12]. To manage excessive secretions in our patient, glycopyrrolate was used, as it is one of the most commonly used antisialogogues [13].

Despite the many challenges in outpatient care caused by spasticity and other manifestations of CP, the recovery from anaesthesia was satisfactory. Anaesthetic management of a patient with CP can be challenging, but to ensure a smooth, uneventful recovery, a comprehensive, meticulous approach should be used to achieve the desired effect.

## Conclusions

We conclude that the anaesthetic management of our patient with SQCP appeared challenging, but with thorough PAC, strategic management of the obstacles, such as proper positioning, decision of using an airway, and managing the oral secretions, helped overcome the possible complications during the perioperative period. We propose a coordinated effort between healthcare providers and anticipate potential challenges and their resolution to achieve safe and successful anaesthetic outcomes.
